# Facile One-pot Transformation of Iron Oxides from Fe_2_O_3_ Nanoparticles to Nanostructured Fe_3_O_4_@C Core-Shell Composites via Combustion Waves

**DOI:** 10.1038/srep21792

**Published:** 2016-02-23

**Authors:** Jungho Shin, Kang Yeol Lee, Taehan Yeo, Wonjoon Choi

**Affiliations:** 1 School of Mechanical Engineering, Korea University (BK21^+^), Seoul, Korea, 136-701.

## Abstract

The development of a low-cost, fast, and large-scale process for the synthesis and manipulation of nanostructured metal oxides is essential for incorporating materials with diverse practical applications. Herein, we present a facile one-pot synthesis method using combustion waves that simultaneously achieves fast reduction and direct formation of carbon coating layers on metal oxide nanostructures. Hybrid composites of Fe_2_O_3_ nanoparticles and nitrocellulose on the cm scale were fabricated by a wet impregnation process. We demonstrated that self-propagating combustion waves along interfacial boundaries between the surface of the metal oxide and the chemical fuels enabled the release of oxygen from Fe_2_O_3_. This accelerated reaction directly transformed Fe_2_O_3_ into Fe_3_O_4_ nanostructures. The distinctive color change from reddish-brown Fe_2_O_3_ to dark-gray Fe_3_O_4_ confirmed the transition of oxidation states and the change in the fundamental properties of the material. Furthermore, it simultaneously formed carbon layers of 5–20 nm thickness coating the surfaces of the resulting Fe_3_O_4_ nanoparticles, which may aid in maintaining the nanostructures and improving the conductivity of the composites. This newly developed use of combustion waves in hybridized nanostructures may permit the precise manipulation of the chemical compositions of other metal oxide nanostructures, as well as the formation of organic/inorganic hybrid nanostructures.

Iron oxides, compounds of iron and oxygen, are among the most abundant metal oxides on earth. Various crystal structures and different combinations of chemical compounds generate unique characteristics in iron oxide species, which are useful in many applications as heterogeneous catalysts^1^,^2^,^3^, pigments^4^, magnetic recording devices^4^, and biomedical applications^5^,^6^,^7^. In the iron oxide family, Fe_2_O_3_ and Fe_3_O_4_ have been the most extensively investigated because of the stability of the materials in general environments^8^,^9^. α-Fe_2_O_3_ and γ-Fe_2_O_3_ are used in geochemistry, red-brown pigments, recording media, and catalysts^10^,^11^,^12^. Development of Fe_3_O_4_ has focused on utilizing the magnetic properties for magnetic devices and sensors, magnetic resonance imaging, ferrofluids, and spintronic devices^13^,^14^,^15^,^16^.

Recently, mesoporous Fe_3_O_4_ has attracted attention in energy conversion and storage research, with uses including battery electrodes^17^,^18^,^19^, capacitor electrodes^20^, and catalysts for photochemical conversion^21^,^22^, because the material possesses large surface area, tunable pore structure, good electrochemical properties, and high stability^23^. However, pure mesoporous Fe_3_O_4_ lacks electrical conductivity among micro- and nanostructures, and causes structural instability and side reactions during repeated electrochemical cycling. As an alternative, carbon-coated Fe_3_O_4_ has been explored to reinforce the deficiencies of the oxide and to protect it from oxidation^20^. The carbon layer can improve the electrical conductivity and stability of micro- and nanostructured Fe_3_O_4_, especially in electrochemical applications. Nanorods, nanowires, and nanospindles of carbon-coated iron oxides have shown enhanced electrochemical performances compared to pure iron oxides^20^,^24^.

The preparation methods for Fe_3_O_4_ nanomaterials and carbon coatings are a significant concern in optimizing the functions of the prepared materials for specific target applications^25^. Micro- and nanostructured Fe_3_O_4_ are generally prepared by microemulsion method^26^,^27^, the thermal decomposition of organometallic compounds^28^,^29^, chemical co-precipitation^30^, hydrothermal method^31^,^32^, or sol-gel method^16^,^33^. These synthesis processes for Fe_3_O_4_ often require high-temperature conditions or have long processing times of several hours at the least. In many cases, the reduction step in Fe_3_O_4_ synthesis uses high-temperature annealing and reducing gases. Furthermore, the formation of the outer carbon layer is generally limited to one of a few methods, such as chemical vapor deposition^34^ and pyrolysis of polymers^35^,^36^. However, this additional processing increases the complexities of production, and time consumption, and production cost. Therefore, the development of a one-step fast fabrication method for carbon-coated Fe_3_O_4_ could facilitate applications in the many fields utilizing Fe_3_O_4_ nanomaterials, especially for electrochemical applications.

In this work, we present a newly developed facile one-pot processing method that transforms iron oxides from Fe_2_O_3_ nanoparticles to nanostructured Fe_3_O_4_@C core-shell composites via combustion waves (Fig. 1). Hybrid composites of nanostructured Fe_2_O_3_ and nitrocellulose were fabricated by impregnating porous films composed of Fe_2_O_3_ nanoparticles with collodion. One-directional, self-propagating combustion waves were realized in the hybrid composites; these waves simultaneously induced the dynamic transformation of Fe_2_O_3_ nanoparticles to Fe_3_O_4_ nanoparticles by fast reduction and formed carbon coatings on the resulting Fe_3_O_4_ nanostructures. After the combustion waves passed through the porous Fe_2_O_3_ nanoparticle films, nanostructured Fe_3_O_4_@C core-shell composites were collected as reaction products. The combustion waves were produced in the hybrid composite of metal oxides and chemical fuels in an open-air environment, without requiring additional supplied gas, vacuum facilities, or furnaces. Moreover, the process was completed no more than a few seconds for porous structures on the cm scale. Therefore, combustion waves in such hybridized structures can be used to fabricate nanostructured Fe_3_O_4_@C core-shell composites, which are useful for many electrochemical applications. The dynamic transformation of oxidation states and the formation of the carbon coating on the metal oxides nanostructures via combustion waves may also be applied to the precise manipulation of other metal oxides, as well as to the formation of organic/inorganic hybrid structures.

## Results and Discussion

### Combustion Waves in Hybrid Composites of Iron Oxides and Nitrocellulose

**Figure 2:**
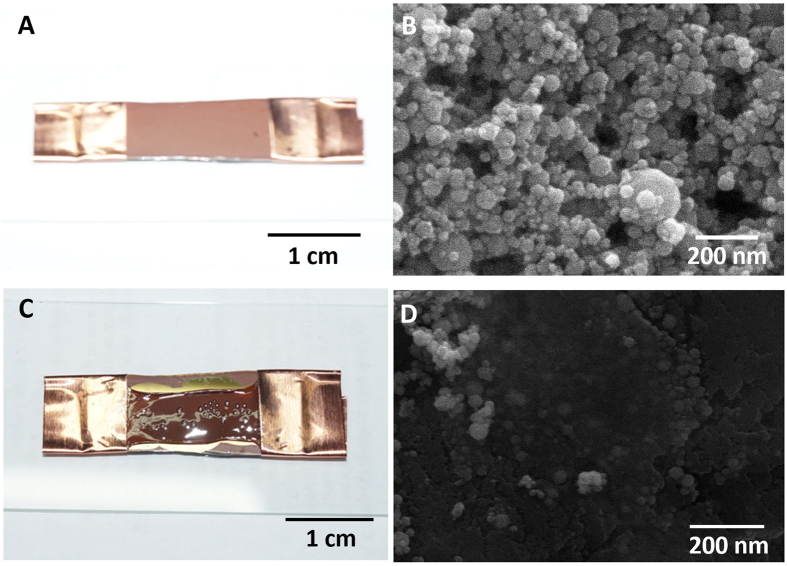
Hybrid composite of Fe_2_O_3_ nanoparticles and nitrocellulose. (**A**) A cm-scale photograph and (**B**) SEM image of the thin Fe_2_O_3_ nanoparticle film on a silicon wafer. (**C**) A cm-scale photograph and (**D**) SEM image of the hybrid composite of Fe_2_O_3_ nanoparticles and nitrocellulose.

Thin films of Fe_2_O_3_ nanoparticles were drop-cast on silicon wafers: Fe_2_O_3_ nanoparticles dispersed in a 5 mg/mL solution with deionized water was dropped onto the silicon wafer. After annealing at 100 °C, the resulting cm-scale film composed of Fe_2_O_3_ nanoparticles appears reddish-brown on the wafer, as shown in Fig. 2A. The average thickness of Fe_2_O_3_ thin films was about 3.6 μm (Fig. S1). The copper tapes on both sides of the film maintain the stability and adhesion of the film on the wafer. SEM examination confirms the inner structures present in the prepared film (Fig. 2B). The diameters of most Fe_2_O_3_ nanoparticles range from 20 nm to 50 nm. The rounded particle shapes and the annealing process, which removed solvents and residue, create highly porous percolation networks of Fe_2_O_3_ nanoparticles in the film.

The hybrid composite of Fe_2_O_3_ nanoparticles and nitrocellulose was fabricated by wet impregnation (Fig. 2C). Collodion was dropped on the top surface of the thin film of Fe_2_O_3_ nanoparticles, permeating the film by filling the porous percolation networks as the solvents evaporated at room temperature. The 95 μl-collodion (5%-nitrocellulose) per unit area (1 cm^2^) completely filled the porous structure which was formed by networks of 5.26 μg-Fe_2_O_3_ particles. Simultaneously, the capillary force within the porous structures induced shrinkage of the original films during the solvent evaporation. After evaporation was complete, the remaining nitrocellulose surrounds the nanoparticles and fills the pores of the films, as shown in Fig. 2D. During the fabrication process of the hybrid composite, the Fe_2_O_3_ films experienced expansion and contraction due to infiltration and evaporation of solvents in collodion. The copper tapes were used as fixing parts to maintain its original shape. The original sizes, shapes, and chemical compositions of the Fe_2_O_3_ nanoparticles are maintained. In this hybrid structure, interfacial boundaries between individual Fe_2_O_3_ nanoparticles and nitrocellulose are necessary, because the direct contact of combustion waves with the surface of each Fe_2_O_3_ nanoparticle is required for the phase transformation of the entire nanostructure, as well as the formation of the carbon coating layer.

**Figure 3:**
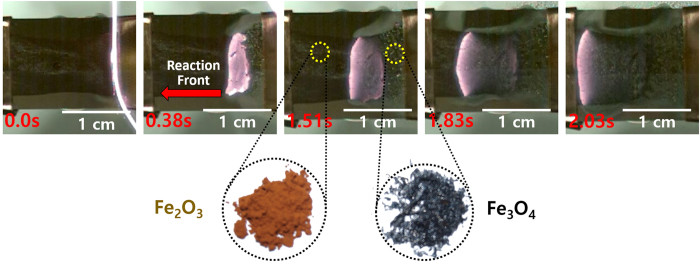
Real-time photographs of the reaction front propagation via combustion wave. The left side of the reaction front is the unreacted regime, composed of Fe_2_O_3_ nanoparticles and chemical fuels, while the right side shows the reacted region composed of Fe_3_O_4_ nanoparticles only. The inset images depict the nanoparticle species before (left, Fe_2_O_3_) and after (right, Fe_3_O_4_) the passage of the combustion wave.

The combustion wave was derived by igniting the nitrocellulose at one end of the hybrid composite film by the resistance heating method. A heated tungsten wire gently touched the film, and the launched reaction developed as the self-propagating combustion wave moved through the percolation networks of Fe_2_O_3_ nanoparticles, without further heat energy applied (Fig. 3). While the combustion wave propagates, a direct color change from reddish-brown to dark-grey is observed in the unreacted and reacted regimes, as shown in the inset of Fig. 3. This provides clear evidence of the structural-chemical phase transformation of the Fe_2_O_3_ nanoparticles via the combustion wave.

**Figure 4:**
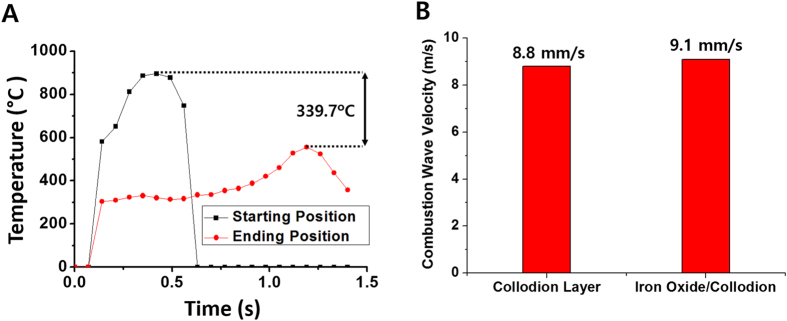
Reaction temperature and velocity of combustion waves. (**A**) Surface temperature profile of the hybrid composite of iron oxides and chemical fuels. Black and red lines indicate the real-time temperature change at the starting and ending positions, respectively, of the combustion wave. (**B**) Comparison of the propagation velocity of combustion waves in a collodion-only (nitrocellulose) layer and the layer containing a mixture of iron oxide nanoparticles and collodion.

In order to clarify the physicochemical conditions of the phase transformation, the changes in temperature and the reaction velocity during the propagation of the combustion wave were obtained by optical pyrometers and the high-speed camera, respectively (Fig. 4). For the temperature measurement, the two optical pyrometers track the real-time temperature changes at the starting and ending positions of the combustion wave in the thin film of iron oxide nanoparticles (Fig. 4A). At the starting position, the temperature quickly increases, and the maximum temperature initially reaches 900 °C. After the reaction front passes, the temperature decreases in a cooling stage. Meanwhile, at the ending position, the temperature remains in the low-temperature regime. When the self-propagating combustion wave passes through the ending position, it reaches a maximum temperature of ~520 °C. Although local variation of the maximum temperatures exists, the entire Fe_2_O_3_ nanoparticle film is exposed to temperatures between 500–900 °C, sufficient to induce phase transformation^37^. Comparing the reaction velocities between the hybrid composite of iron oxides-nitrocellulose and nitrocellulose alone provides intuitive information on the chemical environment formed by the combustion wave (Fig. 4B). The reaction velocity in the hybrid composite is somewhat faster than that in the nitrocellulose-only layer. The direct supply of oxygen from the metal oxides enhances the velocity of the combustion wave, which consumes the surrounding oxygen^38^. This implies that the Fe_2_O_3_ nanoparticles in this hybrid composite film might lose oxygen from the inner structures to the chemical reaction in the combustion wave. This mechanism might cause the phase transformation by changing the oxidation states of the Fe_2_O_3_ nanoparticles.

### Characterization of Phase Transformation and Carbon Coating Layer

**Figure 5:**
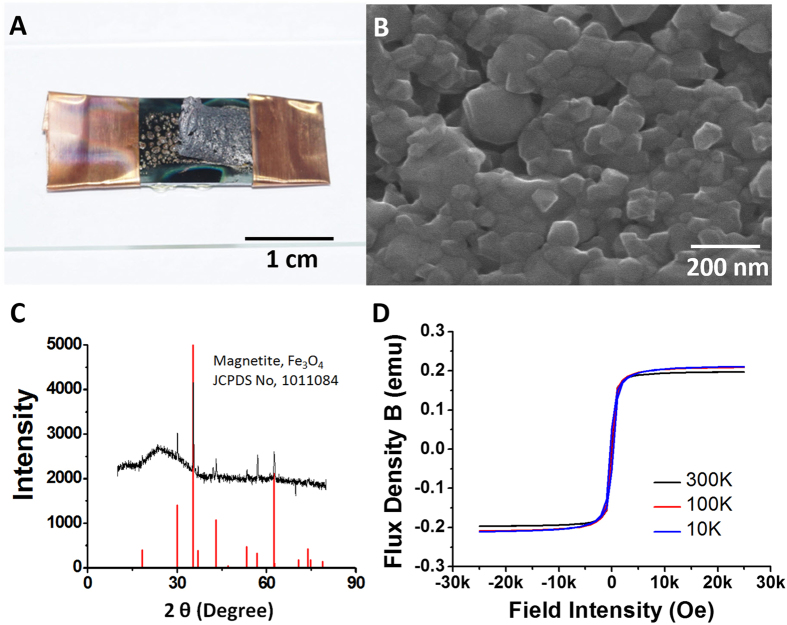
Transformation of iron oxides from Fe_2_O_3_ nanoparticles to nanostructured Fe_3_O_4_@C composites. (**A**) A cm-scale photograph and (**B**) SEM image of the thin film of Fe_3_O_4_ nanostructures after transformation from Fe_2_O_3_ nanoparticles via combustion waves. (**C**) X-ray diffraction (XRD) patterns of iron oxides. (**D**) B-H curve of Fe_3_O_4_ nanoparticles after exposure to combustion wave.

After the phase transformation by the combustion waves, the remaining materials are completely different in color compared to the original Fe_2_O_3_ nanoparticles (Fig. 5A). Before applying the combustion waves, the film of Fe_2_O_3_ nanoparticles shows the reddish-brown color of hematite (Fig. 2A). The hybrid composite maintains this brown color, as shown in Fig. 2C. The chemical-structural changes triggered by the combustion waves included a phase transformation, which accompanies the color change to dark gray. As described above, the combustion waves might provide both the high temperatures and the driving force for reduction by oxygen release for the Fe_2_O_3_ nanoparticles. Under these conditions, most iron oxide materials are transformed to Fe_3_O_4_ (magnetite) nanostructures with a dark gray color^39^. The combustion wave in the hybrid composites may cause the following reactions to occur, changing the oxidation state from Fe_2_O_3_ to Fe_3_O_4_:


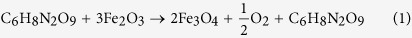



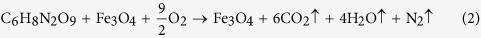


These reactions include the release of oxygen, which accelerates the reaction, as shown in Fig. 4B. To understand the phase transformation, SEM images were obtained of the synthesized Fe_3_O_4_ nanostructures. While the Fe_2_O_3_ nanoparticles were spherical and ~20–50 nm in diameter, the Fe_3_O_4_ nanostructures synthesized by combustion waves show rounded polyhedral shapes with large dimensions ranging from 50 nm to 80 nm. The high temperature and anisotropic pressure waves in combustion may cause aggregation of the Fe_3_O_4_ nanostructures, as well as the morphology changes. The melting points of iron oxides generally exceed 1500 °C, while the surface temperature in the hybrid composites was ~500–900 °C. However, in nanostructured materials, aggregation and morphology changes can occur at much lower temperatures by diffusion and surface boundary variations at the nanoscale^40^.

XRD measurements were performed for greater insight. The peaks from the iron oxide nanostructures synthesized by combustion waves correspond to the (111), (220), (311), (222), (400), (331), (422), (333), (511), (440), (531), (442), (620), (533), and (622) planes of magnetite, Fe_3_O_4_ (JCPDS No. 1011084)^41^. This confirms that combustion waves in open-air conditions could cause the direct phase transformation from Fe_2_O_3_ to Fe_3_O_4_ in a few seconds.

The capacity of magnetization of the synthesized Fe_3_O_4_ by combustion waves were evaluated by the B-H curve of magnetic induction changing with the applied magnetic field (Fig. 5D). Fe_3_O_4_ generally shows magnetization, evaluated by the B-H curve shape. The comparison of the magnetization was conducted using a vibrating-sample magnetometer^42^. As the applied magnetic field increases in strength, larger degrees of magnetism are obtained until the magnetic field approaches 10 kOe. At this point, the magnetism becomes constant. All hysteresis loop widths are very narrow because of the higher-temperature conditions. This curve shapes suggests that the synthesized Fe_3_O_4_ may be superparamagnetic. It shows notable differences in comparison with the curves of pure Fe_3_O_4_. The specific magnetization saturation of the synthesized Fe_3_O_4_ is 0.21 emg at low temperature and 0.19 emg at room temperature. These are one-third of the specific magnetization saturations of pure Fe_3_O_4_^43^, demonstrating that the derived magnetism is relatively small. The magnetization property of Fe_3_O_4_ generally depends on the grain size. Either the large grain size over 100 nm, or the small grain size under 20 nm can provide the strong magnetization, while the grain size in the range of 50 nm and 100 nm relatively shows the weak magnetization^44^. Moreover, the slow cooling rate can affect the magnetization property of Fe_3_O_4_. It is known that thermoremanent magnetization deeply depends on the cooling procedure^45^. The slow cooling rate induces the relatively strong magnetization, whereas the fast cooling rate causes the weak magnetization. The transformation from Fe_2_O_3_ nanoparticles to Fe_3_O_4_ nanostructures by combustion waves experienced the extremely fast cooling rate in a few second with the resulting materials in the range of 50 nm and 80 nm, and it may turn out the weak magnetization^46^.

**Figure 6:**
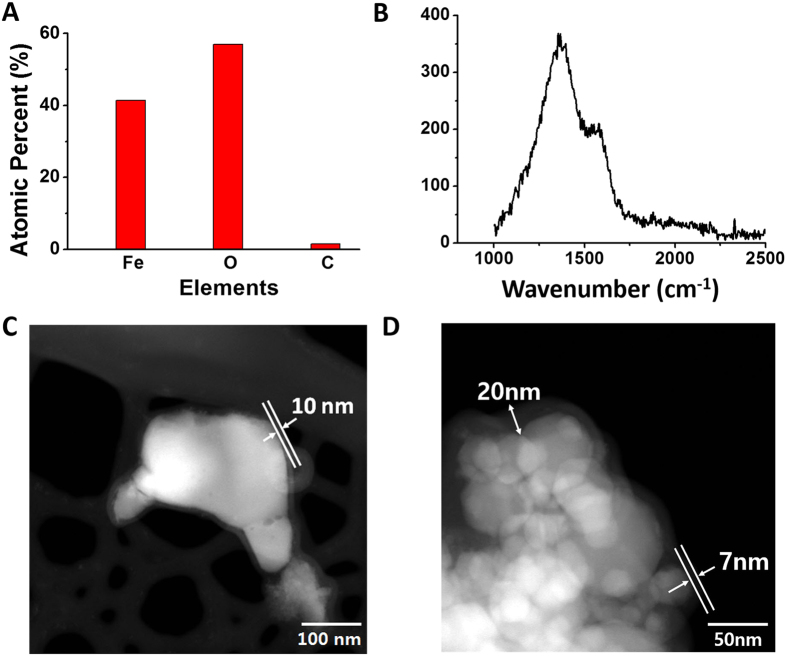
Carbon coating layer in nanostructured Fe_3_O_4_@C composites. (**A**) Atomic percentages of Fe, O, and C from EDX analysis. (**B**) Raman spectrum using 532-nm laser excitation of the synthesized Fe_3_O_4_ nanostructures. (**C**) Extended TEM image of Fe_3_O_4_@C nanostructures. (**D**) Higher-magnification TEM image of a group of Fe_3_O_4_@ C nanostructures.

For further characterization of the chemical compositions of the synthesized Fe_3_O_4_ nanostructures, EDX was conducted. Three different atomic species of iron, oxygen, and carbon remain as the main components after the propagation of the combustion wave (Fig. 6A). The atomic percentages are 41, 57, and 2%, respectively. It was previously demonstrated that solution combustion synthesis could form carbon layers around synthesized materials^47^,^48^. In the combustion of the hybrid composite films of iron oxide and nitrocellulose, the chemical formulas listed above assume perfect combustion conditions. However, in reality, remaining carbon layers exist from the non-combusted carbonaceous chemical fuel. Raman spectroscopy was used to elucidate the detailed properties of the carbon layers in the synthesized materials. The D and G bands are broad, and the Raman peak is located from 1300 cm^−1^ to 1600 cm^−1^ 49, denoting the scattering spectrum of glassy carbon (Fig. 6B). The EDX data and the Raman spectrum confirm that the Fe_3_O_4_ nanostructures definitely have carbon layers after the completion of combustion.

**Figure 7:**
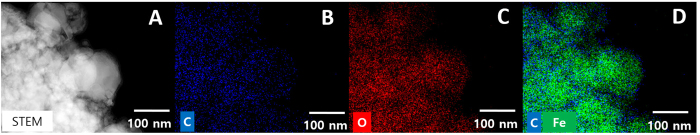
EDX mapping of the Fe_3_O_4_@C nanostructures. (**A**) STEM image, (**B**) carbon atoms, (**C**) oxygen atoms, and (**D**) carbon and iron atoms.

Transmission electron microscopy (TEM) was used to explore the distribution of the carbon layer in the synthesized Fe_3_O_4_ nanostructures. As shown in Fig. 6C,D, the white color represents Fe_3_O_4_, while semitransparent layers are the carbon layers^50^. The dimensions of individual Fe_3_O_4_ nanostructure range from 50 nm to 80 nm, which are similar to the SEM measurements in Fig. 5B. The carbon layer is wrapped around the Fe_3_O_4_ nanostructures, with a thickness of 7–20 nm and an average thickness of 10 nm. The interfacial boundaries of the individual Fe_3_O_4_ nanostructures with the carbon layer in this synthesized Fe_3_O_4_@C are notable. Most surfaces of the Fe_3_O_4_ nanostructures are completely covered by carbon. This implies that the contact between the initial Fe_2_O_3_ nanoparticles and nitrocellulose might be already formed throughout the porous network prior to the combustion wave ignition. After the reaction front passes through the entire film, the interfacial boundaries of the chemical fuel and the metal oxide are converted to carbon layers at the metal oxide surfaces. EDX mapping data clearly shows the core-shell structures of Fe_3_O_4_@C. The high-resolution scanning tunneling electron microscopy (STEM) measurement and corresponding chemical composition analysis are presented in Fig. 7. Figure 7A–D show the high-resolution STEM image and distributions of carbon, oxygen, and carbon-iron pairs in the structure. The core structures of the synthesized composites are clearly Fe_3_O_4_ nanostructures, as shown in Fig. 7C,D. The shell structures of ~10 nm in thickness are recognized as carbon layers, represented by the blue color in Fig. 7B,D. The synthesized Fe_3_O_4_@C composites were stable in open-air conditions. Typical Fe_3_O_4_ nanostructures are easily oxidized to Fe_2_O_3_ nanostructures because of the high reactivity of Fe_3_O_4_ and the large surface area of nanomaterials. However, with the carbon layer formed by the combustion wave, the Fe_3_O_4_@C composites maintain the original structures with respect to size, shape, thickness of carbon layer, and chemical compositions without oxidation for a period of one month.

### Facile One-Pot Phase Transformation with Carbon Coating

To understand the detailed conditions and mechanisms of the phase transformation of metal oxides in the hybridized structure, two control experiments were conducted. In one, the environmental conditions were varied without fast combustion, while the other one removed the interfacial boundaries of the Fe_3_O_4_ nanoparticles and nitrocellulose. In the first experiment, high-temperature annealing at 740 °C, approximately the average surface temperature in the combustion wave, was performed for 3 h on the thin reddish-brown film of Fe_2_O_3_ nanoparticles. This provided high-temperature conditions with a sufficient supply of thermal energy^38^,^51^ without creating the chemical environment produced by combustion waves inside the thin film. Interestingly, no color change was observed after annealing, and no phase transformation occurred. Despite 3 h annealing, the absence of the reducing agent prohibited the phase transformation. An SEM image obtained after annealing is shown in Fig. S2. The Fe_2_O_3_ nanoparticles are somewhat aggregated, but the overall shape of the final aggregate structure differs from that produced by combustion waves. Some structural growth occurred along the length of the film. The XRD peaks from the material after the annealing process (Fig. S2) correspond to the (220), (311), (400), (422), and (440) planes of Fe_2_O_3_ (JCPDS No. 01-077-9927). This measurement confirms that no change of oxidation states occurred. Therefore, both high temperatures and reduced oxygen concentration by the combustion waves are necessary to cause the direct phase transformation of Fe_2_O_3_ nanoparticles. Combustion waves in the hybridized structure can control the oxygen concentration surrounding the iron oxide nanostructures, which dominates the phase transformation of the core structures.

Another control experiment investigated the roles of the interfacial boundaries between the metal oxide and the chemical fuel. For this purpose, layered composites containing a chemical fuel layer (top) and a thin film of Fe_2_O_3_ nanoparticles (bottom) were fabricated, rather than a hybrid composite with interfacial boundaries around the nanostructures. Collodion was poured into a petri dish and kept at room temperature for 30 min to obtain solidified nitrocellulose. This was placed on top of the Fe_2_O_3_ thin film, and silver paste at both ends was used to fix the two layers into one structure. Then, the combustion of nitrocellulose was launched on the Fe_2_O_3_ thin film; the structural-chemical status of the remaining iron oxide was examined by SEM analysis (Fig. S3). In comparison with the SEM image of the Fe_2_O_3_ film layer before combustion (Fig. 2B), no transition is observed, and the original structures are preserved. This proves that the interfacial boundary between individual nanostructures and the chemical fuel is required to complete the phase transformation from Fe_2_O_3_ to Fe_3_O_4_, because the combustion wave along the micro- and nanostructures provide oxygen release as well as a sudden increase in temperature.

**Figure 8:**
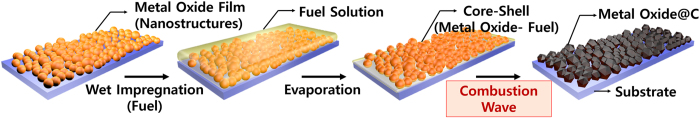
Detailed steps of facile one-pot transformation of nanostructured Fe_3_O_4_@C core-shell composites from Fe_2_O_3_ via combustion wave.

Based on the material analysis, our understanding of combustion waves, and the control experiments, the mechanism for phase transformation and carbon coating in the hybridized structure by the combustion wave is summarized in Fig. 8. The metal oxides must be suitably dispersed on the substrate, forming a highly porous percolation network to promote the infiltration of chemical fuel. Chemical fuel dissolved in organic solvents penetrates the percolation networks. After the evaporation of the solvents, the individually core-shell packed structures of nanostructured metal oxides and chemical fuels are stably formed as the interfacial boundaries for the path of the combustion waves. Finally, the self-propagating combustion wave is sustained such that it passes through the entire interfacial boundary to release oxygen from the core materials to form the carbon layer at the interface. This facile one-pot transformation by combustion waves in hybrid composites of chemical fuels and core materials could be applied to the transformations of other metal oxides and the synthesis of ceramics, as well as providing a general strategy for the formation of a carbon coating layer on nanostructured materials. This reaction is completed within a few seconds without a costly setup, because it is performed in open-air conditions. Therefore, further development of the combustion wave method in this work might lead to the widespread use of low-cost, high-speed synthesis of micro- and nanostructured materials.

## Conclusions

In summary, we performed a facile one-pot transformation of iron oxides from Fe_2_O_3_ nanoparticles to nanostructured Fe_3_O_4_@C core-shell composites via combustion waves. Hybrid composites using Fe_2_O_3_ nanoparticles as core materials and nitrocellulose as chemical fuel were designed and fabricated by a simple wet impregnation method. Self-propagating combustion waves were sustained to pass through the interfacial boundaries in this structure between the Fe_2_O_3_ nanostructures and the nitrocellulose. Because the combustion waves induced the exposure to rapidly increased temperatures in very short timespans, as well as oxygen release from the inner structures, reddish-brown Fe_2_O_3_ nanoparticles were quickly transformed to dark gray Fe_3_O_4_ nanostructures. The remaining Fe_3_O_4_ nanostructures were surrounded by a carbon coating layer, which improved the structural-chemical stability of the synthesized Fe_3_O_4_ as well as the conductivity of the nanostructures. The phase transformation and subsequent carbon coating via combustion wave have various advantages for both material processing and applications. The process is one-step, fast, and large in scale, without high-cost or bulky equipment, since the combustion is completed quickly under atmospheric conditions. To cause the same transformation of metal oxides and formation of carbon coating, wet chemistry reactions or long annealing processes with controlled environments followed by CVD are required. Propagating combustion waves in a hybrid composite of nanostructured materials and chemical fuel may provide one route to overcome these limitations. The technique could be applied to the mass production of organic-inorganic hybrid nanostructures for energy conversion and storage research fields. The further development of this combustion wave method has high potential for the processing and fabrication of nanoscale materials.

## Methods

### Chemicals

Fe_2_O_3_ nanopowders (diameter ≤ 50 nm) were purchased from Sigma-Aldrich. Collodion (5% nitrocellulose, C_6_H_8_N_2_O_9_, in 3:1 dimethylether:EtOH) was purchased from Kanto. All reagents were used as received without purification.

### Fabrication of Fe_2_O_3_ films

Fe_2_O_3_ nanopowders were dissolved in deionized water for a 5 mg/mL solution. The prepared solution was sonicated for 30 min to ensure uniform dispersion. The solution was drop-cast to form a thin film of Fe_2_O_3_ on a silicon wafer. To remove residue and improve the quality of the film, it was annealed for 1 hour at 100 °C. This formed a thin film composed of Fe_2_O_3_ nanoparticles on the silicon wafer.

### Hybrid Composite of Fe_2_O_3_ Nanoparticles and Nitrocellulose

Hybrid composites, which were packing structures composed of Fe_2_O_3_ nanoparticles and nitrocellulose, were fabricated by wet impregnation. Collodion was dropped onto the thin Fe_2_O_3_ nanoparticle film and it permeated into the porous structures with chemical fuel at room temperature. The infiltration of collodion was completed in a few minutes. After drying, the resulting material was a hybrid composite of Fe_2_O_3_ nanoparticles and nitrocellulose in a thin film. Because the nitrocellulose made direct contact with the surfaces of the Fe_2_O_3_ nanoparticles, the mixture could be described as layered core-shell structures Fe_2_O_3_@nitrocellulose on the silicon wafer. In order to maintain the original shape of the film during the drying process, copper tapes were fixed on both sides of the Fe_2_O_3_ film.

### Propagation of Combustion Waves

Combustion waves were initiated by resistance heating using tungsten wire at the leading edge of the hybrid composite. The percolation network of micro- and nanostructured Fe_2_O_3_ and nitrocellulose guided the combustion waves in one direction. A high-speed CCD camera (Phantom V7.3-8GB color camera) with a microscopic lens (Macro 105 mm, f/2.8D, Nikon) recorded the propagation of the reaction front at a rate of 5000 frames/s, which could be converted to the reaction velocity. While the combustion waves existed, two optical pyrometers, a Raytek MM1MHCF1L and a Raytek MM2MLCF1L, measured the real-time temperatures of the films at the starting and ending positions of the chemical reactions, respectively. The first pyrometer measured the spectral response at the 1-μm position with a semiconductor photodetector in the temperature range of 560–3000 °C, while the second pyrometer measured the spectral response at the 1.6-μm position with a semiconductor photodetector in the temperature range of 300–1100 °C.

### Characterization of Iron Oxides Before and After Exposure to Combustion Waves

Diverse methods were implemented for material characterization, permitting a detailed comparison of the iron oxides before and after the propagation of combustion waves. These included scanning electron microscopy (SEM) images, energy dispersive X-ray spectroscopy (EDX) line profile data from a field-emission SEM (FEI, Model Quanta 250 FEG; Jeol, Model JSM-6701F), transmission electron microscope (TEM) images and EDX mapping (FEI, Talos F200 X), Raman spectroscopy (Horiba Jobin Yvon, LabRAM ARAMIS IR^2^ spectrometer), and X-ray diffraction (XRD) patterns (Rigaku, SmartLab). Raman spectra were measured with a 532-nm diode laser as an excitation source. XRD patterns were measured in the 2θ mode at a scan speed of 2°/min. The magnetic properties were measured through the B–H curve for magnetic flux and magnetic field strength (MPMS–7, Quantum Design, USA).

## Additional Information

**How to cite this article**: Shin, J. *et al.* Facile One-pot Transformation of Iron Oxides from Fe_2_O_3_ Nanoparticles to Nanostructured Fe_3_O_4_@C Core-Shell Composites via Combustion Waves. *Sci. Rep.* **6**, 21792; doi: 10.1038/srep21792 (2016).

## Supplementary Material

Supplementary Information

## Figures and Tables

**Figure 1:**
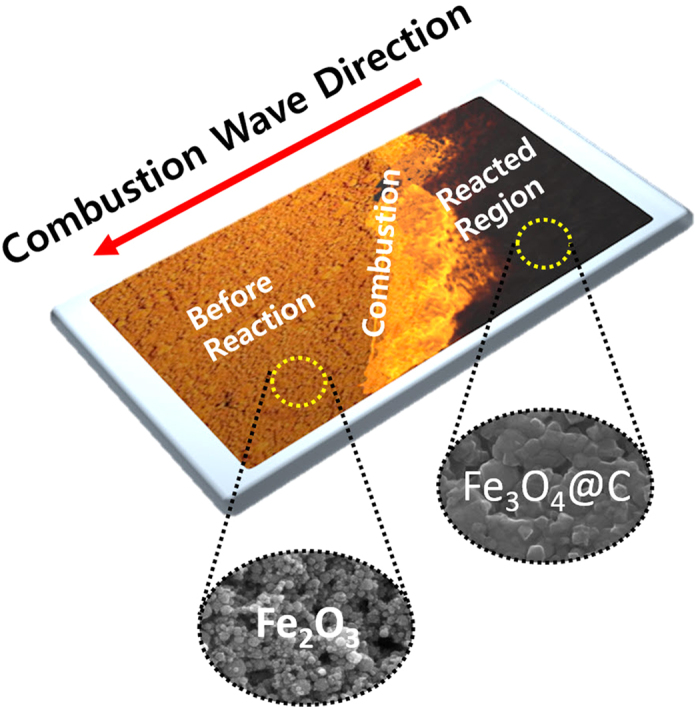
Scheme of one-step phase transformation from Fe_2_O_3_ to Fe_3_O_4_@C via combustion wave in the hybrid composite of Fe_2_O_3_ nanoparticles and chemical fuel.
