# Supplementary material for: Facile One-pot Transformation of Iron Oxides from Fe2O3 Nanoparticles to Nanostructured Fe3O4@C Core-Shell Composites via Combustion Waves
Source: Sci Rep. 2016 Feb 23;6:21792. doi: 10.1038/srep21792 (PMC4763259; doi:10.1038/srep21792)
Supplement: Supplementary Information [file srep21792-s1.pdf]

[**Supporting information**]

Facile One-pot Transformation of Iron Oxides from Fe_2O_3 Nanoparticles to Nanostructured Fe_3O_4@C Core-Shell Composites via Combustion Waves

Jungho Shin, Kang Yeol Lee, Taehan Yeo, and Wonjoon Choi*

School of Mechanical Engineering, Korea University (BK21$^+$), Seoul, Korea, 136-701

*Author to whom correspondence should be addressed: Wonjoon Choi: e-mail: *wojchoi@korea.ac.kr*, Phone: +82 2 3290 5951, Fax: +82 2 926 9290.

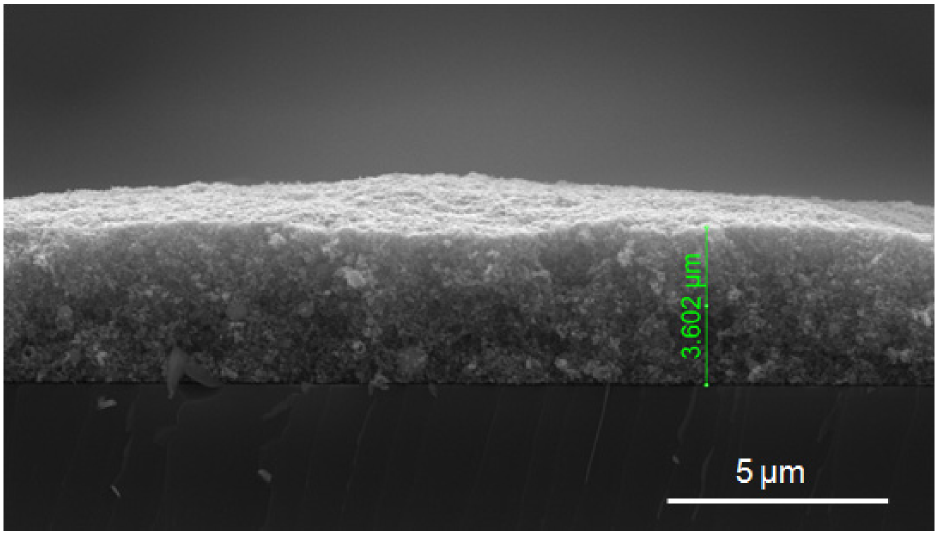

Figure S1. Cross-sectional SEM image of a Fe_2O_3 film.

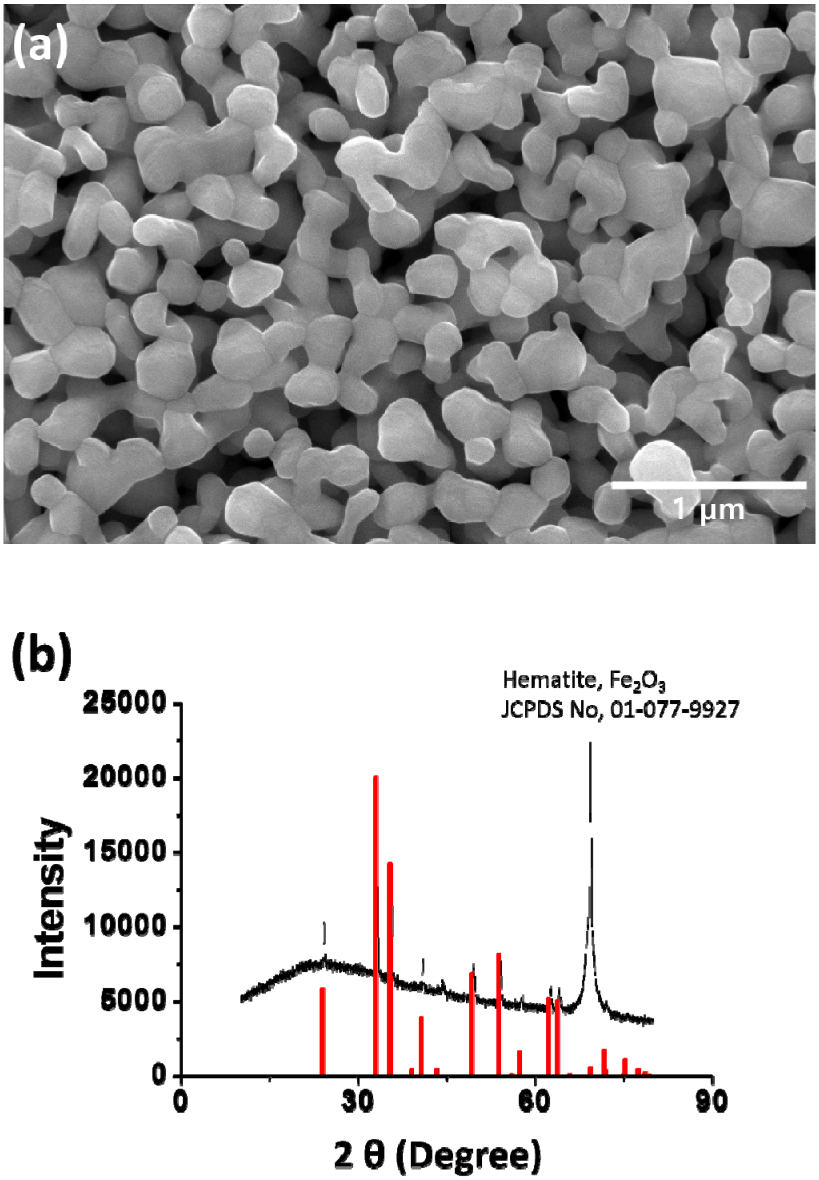

Figure S2. (a) SEM image, and (b) XRD pattern after annealing Fe_2O_3 at 740 °C

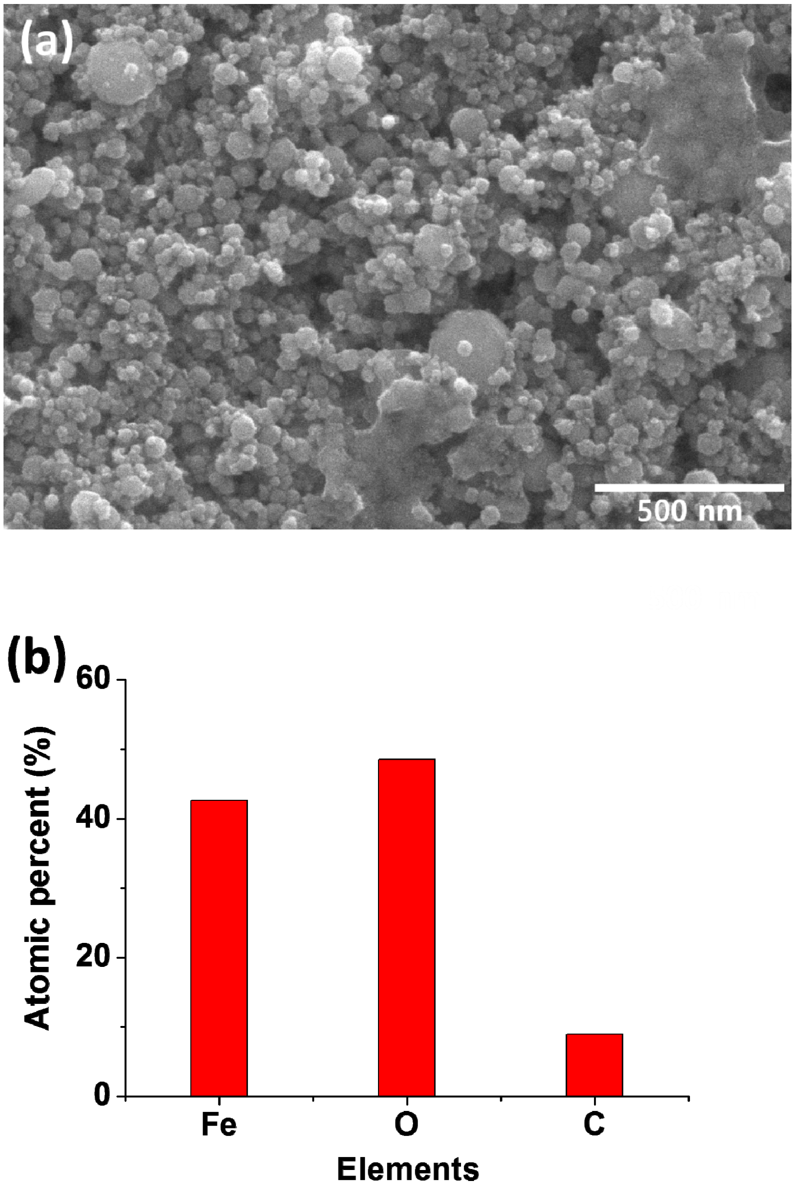

Figure S3. (a) SEM image and (b) Atomic percentages of Fe, O, and C from EDX analysis after combustion wave propagation on a dual layer of unmixed chemical fuel and Fe_2O_3 nanoparticles.

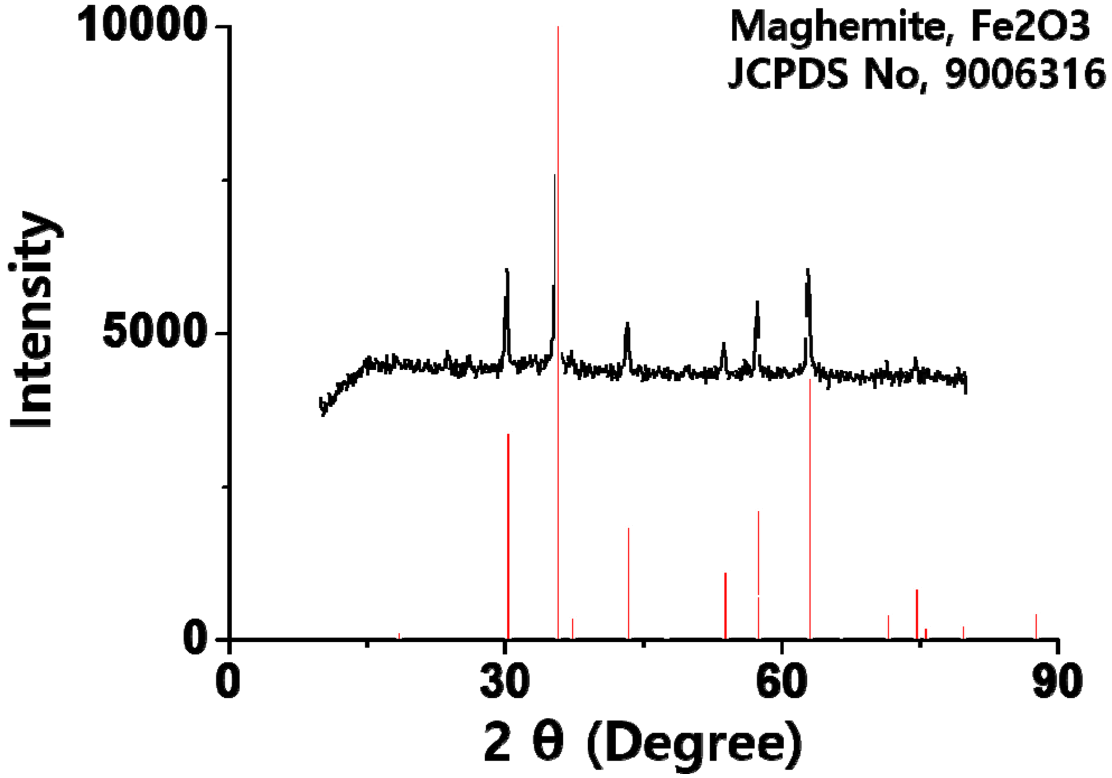

Figure S4. XRD pattern of Fe_2O_3 films before mixing with collodion.
